# Real-world characteristics of trastuzumab deruxtecan-related interstitial lung disease in Japanese patients with breast cancer and gastric cancer

**DOI:** 10.1093/oncolo/oyag340

**Published:** 2026-09-07

**Authors:** Yutaka Fujiwara, Katsunori Oikado, Tomohisa Baba, Masahiko Kusumoto, Shinichi Sasaki, Masahiro Endo, Terufumi Kato, Yasuyuki Kurihara, Yoshinobu Saito, Kunihiro Yagihashi, Masafumi Sata, Yoshinao Sato, Hirotsugu Kenmotsu, Taiki Fukuda, Teppei Yamaguchi, Saiko Isshiki, Hiroshi Wakui, Haruki Kobayashi, Satoshi Ikeda, Motoyasu Kato, Ou Yamaguchi, Toshimi Takano, Ken Kato, Yoshiharu Hiruma, Koji Hirata, Wataru Hashimoto, Kazuyoshi Kuwano

**Affiliations:** Department of Thoracic Oncology, Aichi Cancer Center Hospital, Aichi, 464-8681, Japan; Department of Diagnostic Radiology, Toranomon Hospital, Tokyo, 105-8470, Japan; Department of Respiratory Medicine, Kanagawa Cardiovascular and Respiratory Center, Yokohama-shi, Kanagawa, 236-0051, Japan; Imaging Center, Midtown Clinic East, Tokyo, 107-0052, Japan; Department of Respiratory Medicine, Juntendo University Urayasu Hospital, Urayasu-shi, Chiba, 279-0021, Japan; Division of Diagnostic Radiology, Shizuoka Cancer Center, Shizuoka, 411-8777, Japan; Center for Clinical Research and Trials, Tokyo Metropolitan Cancer and Infectious Diseases Center Komagome Hospital, Tokyo, 113-8677, Japan; Department of Radiology, Kyorin University Hospital, Tokyo, 181-8611, Japan; Department of Respiratory Medicine, Nippon Medical School Musashi Kosugi Hospital, Kawasaki-shi, Kanagawa, 211-8533, Japan; Department of Radiology, St. Marianna University Yokohama Seibu Hospital, Yokohama-shi, Kanagawa, 241-0811, Japan; Department of Internal Medicine, Kyowa Chuo Hospital, Chikusei-shi, Ibaraki, 309-1195, Japan; Department of Diagnostic Imaging, The Cancer Institute Hospital of Japanese Foundation for Cancer Research, Koto, Tokyo, 135-8550, Japan; Division of Thoracic Oncology, Shizuoka Cancer Center, Shizuoka, 411-8777, Japan; Department of Radiology, The Jikei University School of Medicine, Tokyo, 105-8461, Japan; Department of Thoracic Oncology, Aichi Cancer Center Hospital, Aichi, 464-8681, Japan; Department of Radiology, Niiza-Shiki Central General Hospital, Niiza-shi, Saitama, 352-0001, Japan; Division of Respiratory Diseases, Department of Internal Medicine, The Jikei University School of Medicine, Tokyo, 105-8461, Japan; Division of Thoracic Oncology, Shizuoka Cancer Center, Shizuoka, 411-8777, Japan; Department of Thoracic Oncology, Kansai Medical University Hospital, Hirakata-shi, Osaka, 573-1191, Japan; Department of Respiratory Medicine, Juntendo University Graduate School of Medicine, Tokyo, 113-8421, Japan; Department of Respiratory Medicine, Saitama Medical University International Medical Center, Hidaka-shi, Saitama, 350-1298, Japan; Breast Medical Oncology Department, The Cancer Institute Hospital of Japanese Foundation for Cancer Research, Tokyo, 135-8550, Japan; Department of Head and Neck, Esophageal Medical Oncology and Gastrointestinal Medical Oncology, National Cancer Center Hospital, Tokyo, 104-0045, Japan; Department of Medical Safety, Daiichi Sankyo Co., Ltd., Tokyo, 103-8426, Japan; Department of Medical Safety, Daiichi Sankyo Co., Ltd., Tokyo, 103-8426, Japan; Department of Data Intelligence, Daiichi Sankyo Co., Ltd., Tokyo, 140-8710, Japan; Department of Respiratory Medicine, International University of Health and Welfare, Tokyo, 108-8329, Japan

**Keywords:** drug-related side effects and adverse reactions, lung diseases, interstitial, pneumonia, trastuzumab deruxtecan, product surveillance, postmarketing, tomography, X-ray computed

## Abstract

**Background:**

Interstitial lung disease (ILD)/pneumonitis is an important adverse drug reaction associated with trastuzumab deruxtecan (T-DXd). This report comprehensively characterized clinical and imaging features of T-DXd-related ILD, systematically evaluated by an independent ILD Adjudication Committee (AC), in Japanese patients with breast/gastric cancer from all-patient post-marketing surveillance studies.

**Patients and Methods:**

This post hoc analysis evaluated independent ILD AC-adjudicated T-DXd-related ILD cases. Demographics, clinical/radiological features, time to onset, treatment interventions, and outcomes (followed up to 12 months from onset) were analyzed.

**Results:**

Among 2801 T-DXd-treated patients (breast cancer: 1731; gastric cancer: 1070), 421 potential cases were reviewed: 381 were adjudicated as T-DXd-related ILD (breast cancer: 278; gastric cancer: 103). Median age was 65.0 years, 75.1% were female, and 28.3% had a smoking history. ILD occurred throughout observation (including fatal cases ≥12 months post-initiation) with no specific onset window. Time to onset did not differ across CTCAE grades. Organizing pneumonia was the predominant imaging pattern (67.7%), followed by hypersensitivity pneumonitis (14.2%) and diffuse alveolar damage (DAD, 12.6%). Most cases (78.2%) were worst CTCAE Grade 1-2. Grade 5 ILD occurred in 30 patients (7.9%), with DAD the most common pattern (26 patients). Overall recovery was 83.5%. Corticosteroids were used in 54.6% of cases, with 79.3% responding. High-dose corticosteroids and additional immunosuppressants were ineffective in seven DAD cases.

**Conclusions:**

Most T-DXd-related ILD cases were mild/moderate and corticosteroid-responsive; DAD pattern was associated with higher mortality. These findings underscore the importance of vigilant monitoring, early recognition, and prompt corticosteroid treatment to mitigate fatal T-DXd-related ILD.

Implications for practice While trastuzumab deruxtecan (T-DXd) is effective, interstitial lung disease (ILD) remains an important safety concern. Evaluating the first large-scale adjudicated real-world dataset of T-DXd-related ILD in Japan, we found that most cases are mild-to-moderate, predominantly exhibit an organizing pneumonia imaging pattern, and show recovery. However, cases with a diffuse alveolar damage (DAD) pattern have a poor prognosis and can be fatal despite high-dose corticosteroids or immunosuppressants. For clinical practice, these findings underscore that vigilant monitoring, early recognition, and prompt corticosteroid treatment are critical to mitigating fatal T-DXd-related ILD.

## Introduction

Trastuzumab deruxtecan (T-DXd) is an antibody-drug conjugate comprising an anti-human epidermal growth factor receptor 2 (HER2) monoclonal antibody linked by a tetrapeptide-based cleavable linker to a cytotoxic topoisomerase I inhibitor payload.^1^^,^^2^ T-DXd has shown potent anti-tumor efficacy for the treatment of HER2-expressing or mutant cancers.^3–9^

Interstitial lung disease (ILD)/pneumonitis is known as an important adverse drug reaction associated with T-DXd.^10–13^ While clinical trials highlighted concerns regarding T-DXd-related ILD/pneumonitis, less is known about its occurrence in post-marketing use. Recent Japanese all-patient post-marketing surveillance (PMS) studies reported the incidence of T-DXd-related ILD and potential clinical factors of interest in its development among patients with breast^14^ or gastric cancer.^15^ These studies analyzed the overall T-DXd-treated population; however, detailed case-series evaluations of independently adjudicated ILD events were beyond their scope. Therefore, a post hoc case-series analysis focusing specifically on adjudicated T-DXd-related ILD cases with detailed clinical and imaging assessments provides better insight into T-DXd-related ILD characteristics.

In Japan, an independent ILD Adjudication Committee (AC) was established in May 2020, coinciding with T-DXd’s domestic launch, to adjudicate post-marketing ILD cases. This independent ILD AC comprises pulmonologists and radiologists with extensive ILD expertise, alongside breast and gastric cancer specialists. In the all-patient PMS studies, all potential ILD/pneumonitis events that physicians reported in the case report form, where the possibility of ILD could not be ruled out, were centrally reviewed by this independent ILD AC.

We previously reported an interim analysis of 130 cases of T-DXd-related ILD determined by the independent ILD AC, detailing their clinical and imaging characteristics, treatments, and outcomes.^16^ The analysis confirmed that specific ILD imaging patterns, including diffuse alveolar damage (DAD), correlated with greater severity and poorer prognosis, consistent with previous studies.^17^ However, limitations included its interim nature, prioritized review of severe and early-onset cases (resulting in fewer late-onset cases), inclusion of cases with insufficient outcome follow-up, and a small sample size.^16^ Thus, an updated full-scale analysis is warranted to comprehensively assess the patient demographic, clinical, and imaging features of T-DXd-related ILD. Furthermore, information remains limited regarding the ILD treatment status in real-world clinical practice, including the use of corticosteroids or immunosuppressants.

This report aims to comprehensively analyze the clinical and imaging characteristics, treatment interventions, and outcomes of T-DXd-related ILD in Japanese patients with breast or gastric cancer enrolled in PMS studies, based on findings from the independent ILD AC.

## Materials and methods

### Study design and patients

This post hoc analysis included existing and follow-up data from Japanese all-patient PMS studies of T-DXd in breast and gastric cancer, detailed previously.^14^^,^^15^ We focused on cases adjudicated as T-DXd-related ILD by the independent ILD AC, including all events identified by physicians as potential ILD/pneumonitis cases for which site consent for publication was obtained.^14^^,^^15^

All patients with breast cancer (May 25, 2020 [launch date] to November 30, 2021) and gastric cancer (September 25, 2020 [supplemental approval] to December 31, 2021) who initiated T-DXd treatment during these respective periods were enrolled. The safety analysis set included all patients with collected case report forms, excluding the following: those who did not receive T-DXd treatment; initiated T-DXd on or after December 1, 2021 (breast cancer), or after January 1, 2022 (gastric cancer); duplicate registration at the same site; presence/absence of ILD/pneumonitis could not be evaluated (eg, no follow-up after initial visit); off-label T-DXd use; history of T-DXd treatment; and study site declined to provide data for publication. The observation period was up to 18 months for HER2-positive unresectable or recurrent breast cancer and 12 months for HER2-positive unresectable advanced or recurrent gastric cancer. In each PMS study, the observation period ran from T-DXd initiation until the predefined maximum or treatment discontinuation, whichever occurred first. During this period, all reported potential ILD/pneumonitis cases were submitted to the independent ILD AC for review and adjudication. Adjudicated T-DXd-related ILD outcomes were followed for up to 12 months from the reported onset date. If no definitive outcome (recovered/resolved, recovered/resolved with sequelae, or fatal) was confirmed within this window, patients were categorized as recovering/resolving or not recovered/not resolved based on their status at 12 months or at last contact. For cases lost to follow-up, outcomes were determined using the latest clinical or imaging data available after onset. The unknown category was strictly limited to patients for whom no data were provided after ILD onset. Details of the ILD assessment methods and evaluation criteria have been published previously.^16^ This case series includes results up to June 30, 2024.

The all-patient PMS studies were conducted in accordance with Good Post-Marketing Study Practice regulations. As these were PMS studies, written informed consent and institutional review board approval were not required, in accordance with Japanese regulations. The breast and gastric cancer PMS studies were registered at the Japan Registry of Clinical Trials (https://jrct.mhlw.go.jp/en-top) under the identifiers jRCT1080225197 and jRCT2001200001, respectively.

### Assessment of T-DXd-related ILD

Evaluated items for adjudicated cases included patient demographic and clinical characteristics; characteristics of T-DXd-related ILD (for the first ILD event if a patient experienced multiple events), including Common Terminology Criteria for Adverse Events (CTCAE) v5.0^18^ grade at onset (when ILD was first detected) or worst grade by imaging pattern at latest evaluation (determined by the ILD AC based on all available imaging information obtained from the medical institution), time to onset, lung lobe-specific imaging findings at onset, and outcome; corticosteroid responsiveness; and use of immunosuppressants. For fatal cases, the cause of death and its relationship to T-DXd-related ILD were also adjudicated by the independent ILD AC. Lung lobe-specific imaging findings were classified as follows: ground glass opacities (GGO), consolidation, reticulation, and traction bronchiectasis. Regarding the outcome, “proportion of recovery” included the outcomes of recovered/resolved, recovered/resolved with sequelae, or recovering/resolving.

Descriptive statistics were used to summarize patients’ demographic and clinical characteristics, including median (range, quartiles [*Q*]) for continuous data and counts and percentages for categorical data. SAS software version 9.4 (SAS Institute Inc., Cary, NC, USA) was used for statistical analyses.

## Results

### Patients

The flow diagram is shown in Supplementary Figure S1. Overall, 2801 patients were included in the safety analysis set of the all-patient PMS studies conducted in Japan (breast cancer: *n *= 1731, gastric cancer: *n *= 1070). Among them, 421 potential ILD/pneumonitis cases were submitted to the independent ILD AC, and 40 cases were adjudicated as not ILD or not related to T-DXd; reasons included infection, insufficient inspiration or atelectasis, preexisting ILD/interstitial lung abnormalities without worsening, cardiogenic pulmonary edema/congestion, disease progression, radiation pneumonitis, ILD related to other medications, other reasons, and not evaluable. Therefore, 381 cases (breast cancer: *n *= 278, gastric cancer: *n *= 103) were adjudicated as T-DXd-related ILD by the independent ILD AC.

Table 1 summarizes patient demographics. Among the total population with T-DXd-related ILD, the median (range) age was 65.0 (27-86) years, 75.1% were female, and 28.3% of patients were past or current smokers. The median (range) number of prior chemotherapy regimens was 4.0 (2-22).

**Table 1.** Patient demographic and clinical characteristics.

Characteristics		Total *n* = 381	Breast cancer *n* = 278	Gastric cancer *n* = 103
**Sex**	Male	95 (24.9)	4 (1.4)	91 (88.3)
	Female	286 (75.1)	274 (98.6)	12 (11.7)
**Age, years**	Median (range)	65.0 (27-86)	62.0 (27-83)	72.0 (44-86)
	≥65	197 (51.7)	114 (41.0)	83 (80.6)
	≥75	52 (13.6)	19 (6.8)	33 (32.0)
**BMI, kg/m^2^**	<18.5	66 (17.3)	41 (14.7)	25 (24.3)
	18.5 to <25	253 (66.4)	183 (65.8)	70 (68.0)
	25 to <30	53 (13.9)	45 (16.2)	8 (7.8)
	30 to <35	5 (1.3)	5 (1.8)	0 (0)
	≥35	2 (0.5)	2 (0.7)	0 (0)
	Unknown	2 (0.5)	2 (0.7)	0 (0)
**History of smoking**	Yes (past or current smokers)	108 (28.3)	41 (14.7)	67 (65.0)
**ECOG PS**	0-1	359 (94.2)	260 (93.5)	99 (96.1)
	2-4	22 (5.8)	18 (6.5)	4 (3.9)
**Number of prior chemotherapy regimens**	Median (range)	4.0 (2-22)	5.0 (2-22)	3.0 (2-7)
**History of prior ICI**	Yes	58 (15.2)	0 (0)	58 (56.3)
**History of thoracic radiotherapy**	Yes	142 (37.3)	136 (48.9)	6 (5.8)
**CT finding prior to T-DXd treatment evaluated by the independent ILD AC members (radiologists)**	Pre-existing IP^a^	54 (14.2)	31 (11.2)	23 (22.3)
	Radiation pneumonitis or radiation pulmonary fibrosis	94 (24.7)	91 (32.7)	3 (2.9)
	Pleural effusion	67 (17.6)	47 (16.9)	20 (19.4)
	Multiple lung metastasis	140 (36.7)	118 (42.4)	22 (21.4)
	Lymphangitic carcinomatosis	20 (5.2)	19 (6.8)	1 (1.0)
**Prior medical histories reported by physicians**	Respiratory disease^b^	27 (7.1)	18 (6.5)	9 (8.7)
	Heart disease	12 (3.1)	5 (1.8)	7 (6.8)
	History of lung surgery	21 (5.5)	19 (6.8)	2 (1.9)
	Pulmonary thromboembolism	4 (1.0)	1 (0.4)	3 (2.9)
	Autoimmune disease	2 (0.5)	2 (0.7)	0 (0)
	Malignant tumor other than breast or gastric cancer	22 (5.8)	13 (4.7)	9 (8.7)
**Initial T-DXd dose, mg/kg**	Mean ± SD	5.58 ± 0.506	5.35 ± 0.250	6.20 ± 0.503
	Median (range)	5.4 (3.2-6.4)	5.4 (3.2-5.4)	6.4 (4.4-6.4)
	≤3.2	1 (0.3)	1 (0.4)	0 (0)
	>3.2 to ≤4.4	15 (3.9)	11 (4.0)	4 (3.9)
	>4.4 to ≤5.4	277 (72.7)	266 (95.7)	11 (10.7)
	>5.4 to ≤6.4	88 (23.1)	0 (0)	88 (85.4)
	>6.4	0 (0)	0 (0)	0 (0)

Data are shown as counts and percentages unless otherwise stated.

Abbreviations: AC, Adjudication Committee; BMI, body mass index; COPD, chronic obstructive pulmonary disease; CT, computed tomography; ECOG PS, Eastern Cooperative Oncology Group performance status; ICI, immune checkpoint inhibitor; ILD, interstitial lung disease; IP, interstitial pneumonia; SD, standard deviation; T-DXd, trastuzumab deruxtecan.

a Any interstitial shadow on the lung CT scan including chronic fibrotic interstitial pneumonia or interstitial lung abnormalities; not including gravity-dependent opacity.

b Including ILD, pulmonary fibrosis, radiation pneumonitis, COPD, emphysema, asthma, or other respiratory disease.

Among patients with breast cancer, 98.6% were female and 48.9% had a history of thoracic radiotherapy, whereas among those with gastric cancer, 11.7% were female and 5.8% had a history of thoracic radiotherapy. Patients with gastric cancer tended to be older than those with breast cancer (median age 72.0 vs 62.0 years). Smoking history was reported in 65.0% vs 14.7% of patients in the gastric and breast cancer groups, respectively. Prior immune checkpoint inhibitor use was reported in 56.3% of patients in the gastric cancer group and none in the breast cancer group, reflecting differences in standard treatment rather than patient background. In the aggregated case report forms entered by the attending physicians, the prevalence of preexisting or concomitant respiratory disease was 7.1%; however, the independent ILD AC radiologist evaluation identified preexisting interstitial pneumonia findings on computed tomography (CT) scans in 14.2% of patients and radiation pneumonitis or radiation fibrosis findings in 24.7%. The median (range) initial dose of T-DXd was 5.4 (3.2-6.4) mg/kg in the total population, 5.4 (3.2-5.4) mg/kg in the breast cancer group, and 6.4 (4.4-6.4) mg/kg in the gastric cancer group. Most patients in the breast cancer group (95.7%) received an initial T-DXd dose of >4.4 to ≤5.4 mg/kg, and most patients in the gastric cancer group (85.4%) received an initial T-DXd dose of >5.4 to ≤6.4 mg/kg, reflecting the approved dosage for each respective cancer type (5.4 mg/kg for breast cancer and 6.4 mg/kg for gastric cancer).

### Time to onset of T-DXd-related ILD/pneumonitis

The time to onset of initial T-DXd-related ILD by worst CTCAE grade is shown in Figure 1. The median time to onset was 133.0 days, and most cases occurred within the first 6 months. Although more ILD cases occurred early, when more patients remained on treatment and at risk, cases were observed throughout the treatment period. No specific time window for ILD onset was identified. The cumulative number of ILD did not plateau during the observation period.

**Figure 1.**
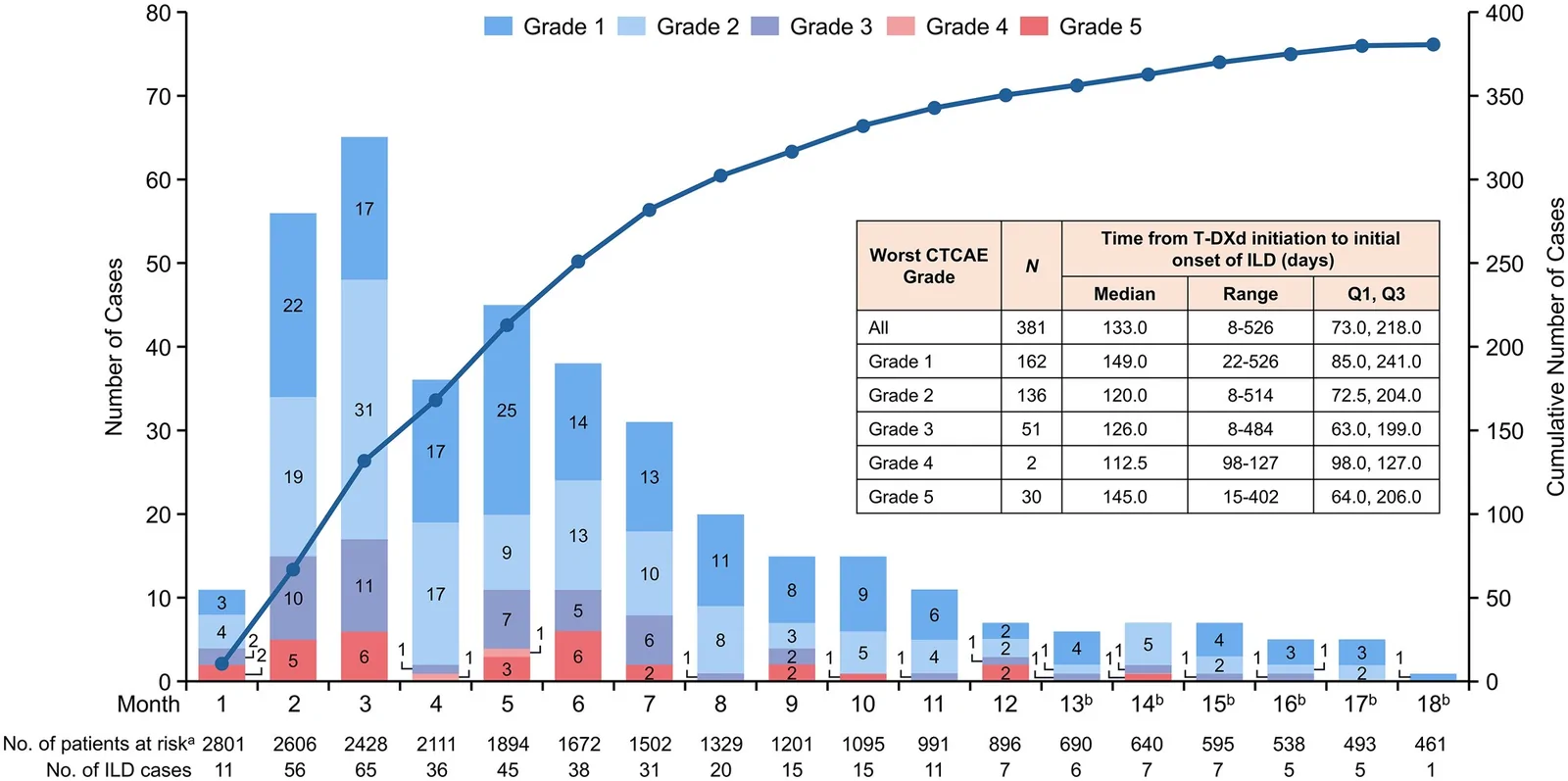
Time to onset of initial T-DXd-related ILD by worst CTCAE grade. Observation period: breast cancer: 18 months, gastric cancer: 12 months. ^a^No. of patients at risk refers to the PMS safety analysis set (*n* = 2801) and is provided for context to aid interpretation of the time-to-onset distribution. ^b^Data for that month include data from only breast cancer PMS studies. Abbreviations: CTCAE, Common Terminology Criteria for Adverse Events; ILD, interstitial lung disease; PMS, post-marketing surveillance; Q, quartile; T-DXd, trastuzumab deruxtecan.

There was no notable difference in the time to onset among CTCAE grades (Figure 1). The median time to onset of initial T-DXd-related ILD was 149.0 days for Grade 1, 120.0 days for Grade 2, 126.0 days for Grade 3, 112.5 days for Grade 4, and 145.0 days for Grade 5 ILD. There were three cases in which ILD developed ≥12 months after T-DXd initiation and resulted in death. For 28.6% of cases (109/381), the independent ILD AC considered that the time of onset was earlier than that recorded by the physicians (median difference: 43.0 days [range: 1-408 days]).

### Imaging pattern and severity of ILD

Table 2 summarizes CTCAE grade at onset stratified by latest imaging pattern. Among the total population with T-DXd-related ILD, the most common imaging pattern was organizing pneumonia (OP) (67.7%, 258/381), followed by hypersensitivity pneumonitis (HP) (14.2%, 54/381) and DAD (12.6%, 48/381). Among the 48 cases showing a DAD pattern in the latest evaluation, 23 were low grade (Grade 1-2) at onset. Overall, the majority of cases were Grade 1 or 2 ILD (60.1% [229/381] and 27.0% [103/381], respectively); 12.6% (48/381) and 0.3% (1/381) cases were Grade 3 and 4 ILD at onset, respectively. Similar imaging patterns were observed between the groups with breast and gastric cancer (Table 2).

**Table 2.** CTCAE grade at onset and worst CTCAE grade stratified by imaging pattern at latest evaluation^a^.

	Any grade	CTCAE grade at onset					Worst CTCAE grade				
		Grade 1	Grade 2	Grade 3	Grade 4	Grade 5	Grade 1	Grade 2	Grade 3	Grade 4	Grade 5
**Imaging pattern total (*n* = 381)**											
** Total**	381 (100)	229 (60.1)	103 (27.0)	48 (12.6)	1 (0.3)	0 (0)	162 (42.5)	136 (35.7)	51 (13.4)	2 (0.5)	30 (7.9)
** DAD**	48 (12.6)	10 (2.6)	13 (3.4)	24 (6.3)	1 (0.3)	0 (0)	0 (0)	3 (0.8)	18 (4.7)	1 (0.3)	26 (6.8)
** HP**	54 (14.2)	28 (7.3)	23 (6.0)	3 (0.8)	0 (0)	0 (0)	24 (6.3)	26 (6.8)	4 (1.0)	0 (0)	0 (0)
** OP**	258 (67.7)	179 (47.0)	61 (16.0)	18 (4.7)	0 (0)	0 (0)	128 (33.6)	101 (26.5)	24 (6.3)	1 (0.3)	4 (1.0)
** NSIP**	10 (2.6)	8 (2.1)	1 (0.3)	1 (0.3)	0 (0)	0 (0)	6 (1.6)	2 (0.5)	2 (0.5)	0 (0)	0 (0)
** Other/indeterminate**	5 (1.3)	1 (0.3)	3 (0.8)	1 (0.3)	0 (0)	0 (0)	1 (0.3)	3 (0.8)	1 (0.3)	0 (0)	0 (0)
** Not evaluated**	6 (1.6)	3 (0.8)	2 (0.5)	1 (0.3)	0 (0)	0 (0)	3 (0.8)	1 (0.3)	2 (0.5)	0 (0)	0 (0)
**Breast cancer (*n* = 278)**											
** Total**	278 (100)	177 (63.7)	69 (24.8)	31 (11.2)	1 (0.4)	0 (0)	125 (45.0)	101 (36.3)	34 (12.2)	1 (0.4)	17 (6.1)
** DAD**	33 (11.9)	6 (2.2)	10 (3.6)	16 (5.8)	1 (0.4)	0 (0)	0 (0)	3 (1.1)	14 (5.0)	1 (0.4)	15 (5.4)
** HP**	36 (12.9)	19 (6.8)	15 (5.4)	2 (0.7)	0 (0)	0 (0)	17 (6.1)	16 (5.8)	3 (1.1)	0 (0)	0 (0)
** OP**	198 (71.2)	144 (51.8)	42 (15.1)	12 (4.3)	0 (0)	0 (0)	102 (36.7)	78 (28.1)	16 (5.8)	0 (0)	2 (0.7)
** NSIP**	4 (1.4)	4 (1.4)	0 (0)	0 (0)	0 (0)	0 (0)	2 (0.7)	2 (0.7)	0 (0)	0 (0)	0 (0)
** Other/indeterminate**	3 (1.1)	1 (0.4)	2 (0.7)	0 (0)	0 (0)	0 (0)	1 (0.4)	2 (0.7)	0 (0)	0 (0)	0 (0)
** Not evaluated**	4 (1.4)	3 (1.1)	0 (0)	1 (0.4)	0 (0)	0 (0)	3 (1.1)	0 (0)	1 (0.4)	0 (0)	0 (0)
**Gastric cancer (*n* = 103)**											
** Total**	103 (100)	52 (50.5)	34 (33.0)	17 (16.5)	0 (0)	0 (0)	37 (35.9)	35 (34.0)	17 (16.5)	1 (1.0)	13 (12.6)
** DAD**	15 (14.6)	4 (3.9)	3 (2.9)	8 (7.8)	0 (0)	0 (0)	0 (0)	0 (0)	4 (3.9)	0 (0)	11 (10.7)
** HP**	18 (17.5)	9 (8.7)	8 (7.8)	1 (1.0)	0 (0)	0 (0)	7 (6.8)	10 (9.7)	1 (1.0)	0 (0)	0 (0)
** OP**	60 (58.3)	35 (34.0)	19 (18.4)	6 (5.8)	0 (0)	0 (0)	26 (25.2)	23 (22.3)	8 (7.8)	1 (1.0)	2 (1.9)
** NSIP**	6 (5.8)	4 (3.9)	1 (1.0)	1 (1.0)	0 (0)	0 (0)	4 (3.9)	0 (0)	2 (1.9)	0 (0)	0 (0)
** Other/indeterminate**	2 (1.9)	0 (0)	1 (1.0)	1 (1.0)	0 (0)	0 (0)	0 (0)	1 (1.0)	1 (1.0)	0 (0)	0 (0)
** Not evaluated**	2 (1.9)	0 (0)	2 (1.9)	0 (0)	0 (0)	0 (0)	0 (0)	1 (1.0)	1 (1.0)	0 (0)	0 (0)

Data are counts and percentages.

Analyses are based on the first ILD event per patient; each patient is counted once. For patients with multiple ILD events, only the first event was included. CTCAE grade at onset is the grade at first detection of the first event.

Other/indeterminate: Radiation recall: *n = *2, non-cardiogenic pulmonary edema: *n = *2, OP and pulmonary edema: *n = *1.

Not evaluated: Although T-DXd-related ILD could not be ruled out based on clinical information, the imaging pattern could not be evaluated as no images were provided: *n = *4, a definitive diagnosis of ILD could not be made because of very slight shadows: *n = *1, an imaging pattern could not be determined due to the worsening of carcinomatous lymphangiosis as a comorbidity: *n = *1.

Abbreviations: CTCAE, Common Terminology Criteria for Adverse Events; DAD, diffuse alveolar damage; HP, hypersensitivity pneumonitis; ILD, interstitial lung disease; NSIP, nonspecific interstitial pneumonia; OP, organizing pneumonia; T-DXd, trastuzumab deruxtecan.

a Imaging pattern at latest evaluation was determined by the ILD Adjudication Committee based on all available imaging information obtained from the medical institution. The time points and number of images vary by case.

Worst CTCAE grade of ILD stratified by imaging pattern at the latest evaluation was also analyzed (Table 2). Among cases with adjudicated T-DXd-related ILD, the majority were Grade 1 (42.5%, 162/381) and Grade 2 (35.7%, 136/381). Grade 5 ILD was observed in 30 patients, representing 7.9% of the adjudicated ILD cohort (30/381). Among these cases, the most common imaging pattern was DAD (26 cases), although four cases with OP pattern were also observed. No notable differences were observed between the groups with breast cancer and gastric cancer.

### Imaging findings at ILD onset by lung lobe

Figure S2 presents imaging findings at ILD onset by lung lobe overall (S2A), stratified by imaging pattern at the latest evaluation (S2B), and by laterality and number of involved lobes (S2C). Of 381 T-DXd-related ILD cases, 377 were analyzed (excluding four lacking onset CT scans). Among the 377 cases, the most common lung lobe-specific imaging finding was GGO (89.4%), followed by consolidation (54.4%), traction bronchiectasis (10.9%), and reticulation (10.6%). No specific trends indicating the imaging findings were more likely to be detected in a particular lobe (Figure S2A). Traction bronchiectasis was present at onset in approximately half of ILD cases with DAD pattern. Generally, DAD and HP were diffuse, with opacities distributed across the entire lung field. The OP pattern showed a high proportion of consolidation along with GGO, resembling the typical features of cryptogenic organizing pneumonia (Figure S2B). Regarding laterality, cases with unilateral involvement or involvement limited to a single lobe were rare; most cases presented with bilateral or multilobar involvement, with abnormal opacities present at onset (Figure S2C).

### Outcomes of T-DXd-related ILD

The overall outcomes of T-DXd-related ILD are shown in Figure 2A. The proportion of recovery (including cases that recovered/resolved, recovered/resolved with sequelae, or were recovering/resolving) was 83.5%. The median (Q1-Q3) time to recovery was 143.0 (63.0-231.0) days. In total, among 381 patients with T-DXd-related ILD, 30 patients died (7.9%). The independent ILD AC adjudicated T-DXd-related ILD as a cause of death in all 30 cases: the sole identified cause in 22, with possible concomitant infection in six, and with cancer progression in two.

**Figure 2.**
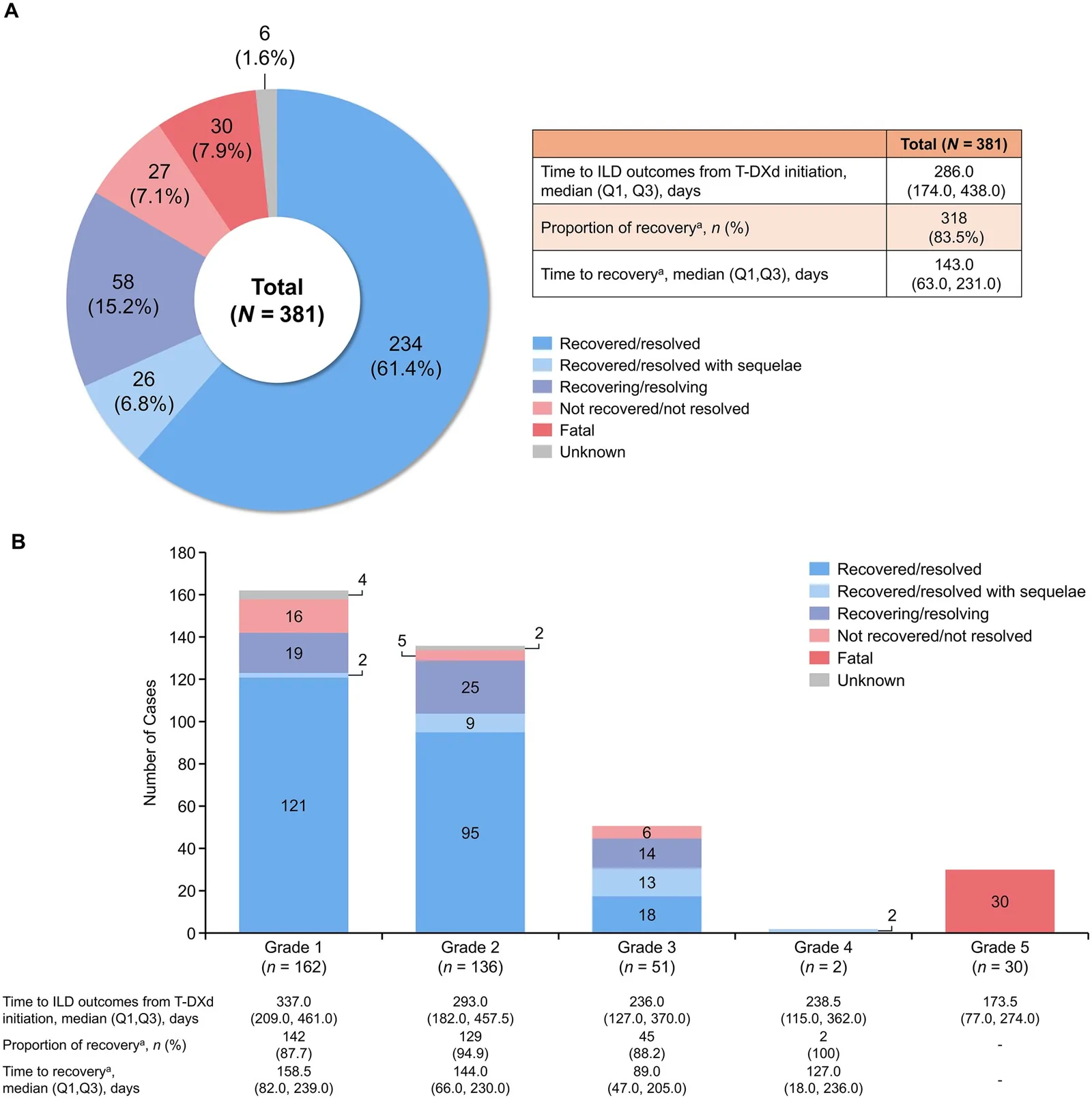
Overall outcomes of T-DXd-related ILD (A), outcomes of T-DXd-related ILD by worst CTCAE grade (B), and outcomes of T-DXd-related ILD by imaging pattern at latest evaluation (C). ^a^“Recovery” refers to patients with outcomes of recovered/resolved, recovered/resolved with sequelae, or recovering/resolving. Abbreviations: CTCAE, Common Terminology Criteria for Adverse Events; DAD, diffuse alveolar damage; ILD, interstitial lung disease; HP, hypersensitivity pneumonitis; NE, not evaluated; NSIP, non-specific interstitial pneumonia; OP, organizing pneumonia; Q, quartile; T-DXd, trastuzumab deruxtecan.

**Figure 2.**
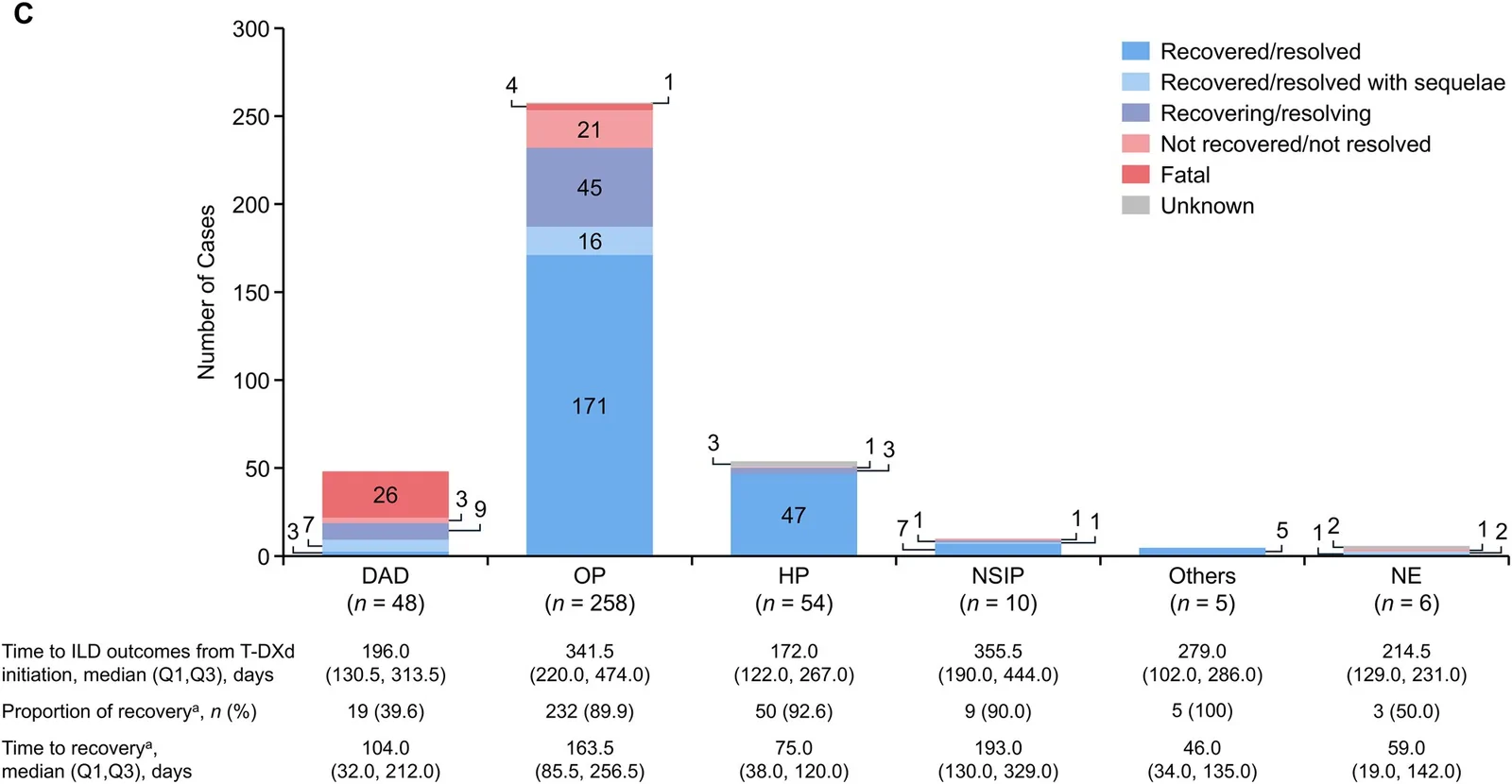
Continued.

The outcomes of T-DXd-related ILD by worst CTCAE grade and by imaging pattern at the latest evaluation are shown in Figure 2B and C, respectively. The proportion of recovery among those with Grade 1, 2, 3, and 4 T-DXd-related ILD was 87.7%, 94.9%, 88.2%, and 100%, respectively (Figure 2B). While the proportion of recovered/resolved cases was high among the low-grade categories, this proportion was relatively lower in patients with Grade 3 T-DXd-related ILD. When stratified by imaging pattern, DAD pattern was associated with higher fatality, resulting in a lower proportion of recovery of 39.6% (Figure 2C). In contrast, other imaging patterns showed higher proportions of recovery (HP pattern: 92.6%, nonspecific interstitial pneumonia [NSIP] pattern: 90.0%, and OP pattern: 89.9%) (Figure 2C).

### Corticosteroid responsiveness

The results of corticosteroid responsiveness are shown in Table 3. Corticosteroids were administered in 208 (54.6%) of the 381 ILD cases in total, in 21 of the 162 cases classified as Grade 1, in 104 of the 136 cases classified as Grade 2, and in all cases classified as Grade 3 or higher. The assessment of corticosteroid responsiveness by the independent ILD AC indicated that the majority of ILD cases (79.3%, 165/208) treated with corticosteroids were considered responsive to the treatment. According to CTCAE worst grade, the proportion of cases with corticosteroid responsiveness was 85.7%, 95.2%, 90.2%, and 100% among Grade 1, 2, 3, and 4 T-DXd-related ILD cases, respectively.

**Table 3.** Corticosteroid responsiveness.

Total (*n* = 381)	Worst CTCAE grade					Total
	Grade 1	Grade 2	Grade 3	Grade 4	Grade 5	
**Adjudicated T-DXd-related ILD**	162 (42.5)	136 (35.7)	51 (13.4)	2 (0.5)	30 (7.9)	381 (100)
**T-DXd-related ILD without corticosteroid treatment**	141 (37.0)	32 (8.4)	0 (0)	0 (0)	0 (0)	173 (45.4)
**T-DXd-related ILD receiving corticosteroids**	21 (5.5)	104 (27.3)	51 (13.4)	2 (0.5)	30 (7.9)	208 (54.6)
**Corticosteroid responsiveness^a^**						
** Yes**	18 (85.7)	99 (95.2)	46 (90.2)	2 (100)	0 (0)	165 (79.3)
** No**	2 (9.5)	2 (1.9)	2 (3.9)	0 (0)	30 (100)	36 (17.3)
** Not evaluated^b^**	1 (4.8)	3 (2.9)	3 (5.9)	0 (0)	0 (0)	7 (3.4)

Data are counts and percentages.

Abbreviations: AC, Adjudication Committee; CTCAE, Common Terminology Criteria for Adverse Events; ILD, interstitial lung disease; T-DXd, trastuzumab deruxtecan.

a Corticosteroid responsiveness was judged by pulmonologists from the independent ILD AC based on the patient’s clinical course, radiological images, and other relevant data for adjudication. The number of patients who received corticosteroid treatment within each CTCAE grade group was used as the denominator for calculating the proportion.

b The independent ILD AC judged the cases to be not evaluable, as neither clinical nor imaging data were provided following the onset of ILD.

### Use of immunosuppressants in T-DXd-related ILD

Among 381 T-DXd-related ILD cases, immunosuppressive agents (cyclophosphamide, cyclosporine, azathioprine, tocilizumab, and tacrolimus) were used in eight cases (Figure S3). The use of these agents for this indication is considered off-label under the Japanese national health insurance system. In seven out of eight cases, the use of these immunosuppressants did not result in clinical improvement. Details of the course and treatments for the eight cases are presented in Figure S3.

## Discussion

This report comprehensively analyzes clinical and imaging characteristics, treatment interventions, and outcomes of T-DXd-related ILD in patients with breast or gastric cancer enrolled in PMS studies conducted in Japan, based on the independent ILD AC. Japanese patients are recognized as being at increased risk for drug-induced ILD, with higher incidence rates of T-DXd-related ILD observed compared to the rest of the world.^10^ Despite these differences, the findings on radiological patterns and clinical features characterized in this report are expected to inform and raise awareness globally, regardless of patient ethnicity. Overall, 421 potential ILD/pneumonitis cases were submitted to the independent ILD AC, and 40 cases were adjudicated as not ILD or not related to T-DXd; 381 cases were adjudicated as T-DXd-related ILD based on a later data cutoff than that used in the previous interim report.^16^ As this case-series analysis focused exclusively on patients who developed T-DXd-related ILD, it did not assess the overall incidence or mortality rates based on the total treated population; these overall rates have been reported in the primary PMS studies.^14^^,^^15^ Overall, the characteristics and clinical features of T-DXd-related ILD observed in the current report were consistent with those reported previously.^10^^,^^16^^,^^17^ The proportion of worst CTCAE Grade 1-2 (mild or moderate cases) T-DXd-related ILD cases in the present report was 78.2%, which aligns with that reported in a pooled analysis of nine clinical trials (77.4%).^10^ The imaging pattern distribution in the present report was 67.7% for OP, 14.2% for HP, 12.6% for DAD, and 2.6% for NSIP. Although the classification of imaging patterns differed slightly, the proportions of OP and DAD patterns were consistent with those observed in clinical trial data.^17^ It is important to note that the 30 fatal cases represent 7.9% (30/381) of the adjudicated ILD cohort evaluated in this case series. This proportion of fatal outcomes among patients who developed ILD is comparable to or even lower than the 14.1% (25/177) reported among ILD cases in the pooled analysis of clinical trials.^10^ Among the 30 fatal cases, 26 (86.7%) exhibited a DAD pattern at the latest evaluation. In our previous interim report, eight of 11 fatal cases (72.7%) also exhibited this pattern.^16^ These findings suggest that the DAD pattern remains prevalent in the majority of fatal cases. The proportion of recovery in the present analysis was 83.5% compared with 76.9% in the previous interim report.^16^ The proportion of patients with corticosteroid responsiveness was 79.3% in the present analysis, indicating that corticosteroid responsiveness remains generally favorable.

The present report substantiates the findings from prior clinical trials and early analyses, further advancing our understanding of T-DXd-related ILD. Most cases were mild or moderate in severity, and a consistent association between imaging patterns and clinical outcomes was observed. Appropriate interventions, such as corticosteroid administration, led to improvement of ILD in many cases. Long-term follow-up showed a decrease in ILD cases with the outcome of recovering/resolving, while cases determined as recovered/resolved increased. However, the overall proportion of recovery remained consistent with previous reports.^16^ Notably, OP is characterized by cases where opacities persist long-term due to fibrosis or scarring, even after discontinuing T-DXd treatment or administering corticosteroids. In such cases, we classified the outcomes as recovered/resolved according to the T-DXd pulmonary toxicity guideline.^19^

At onset, T-DXd-related ILD most commonly presented as bilateral, multilobar abnormalities with GGO as the predominant finding and consolidation observed in approximately half of the patients, while reticulation and traction bronchiectasis were infrequent. Although the right middle lobe showed fewer lesions in absolute number due to its smaller volume, its relative involvement was comparable to other lobes, indicating no preferential lobar predilection. These findings suggest a diffuse inflammatory lung injury affecting the entire lung field from the earliest stages. This profile is consistent with other drug-induced ILD, in which bilateral, diffuse GGO, and OP/NSIP-like patterns are most common, while DAD occurs as a more severe subset.^20–22^ It also resembles immune checkpoint inhibitor- and epidermal growth factor receptor tyrosine kinase inhibitor-related pneumonitis,^23–25^ which typically involves multiple lobes bilaterally. Compared with cytotoxic agents such as bleomycin or methotrexate, T-DXd-related ILD appears to show fewer fibrotic features at onset.^26^^,^^27^

Compared with other lung diseases, T-DXd-related ILD shared the feature of bilateral GGO with viral pneumonias but lacked lower-lobe predominance.^28^^,^^29^ It also differed from community-acquired pneumonia,^30^ carcinomatous lymphangitis,^31^ cardiogenic pulmonary edema,^32^ and radiation pneumonitis.^33^

Differentiating T-DXd-related ILD from non-drug-related lung diseases based on CT findings alone is challenging. Overlapping radiographic patterns may occur, particularly in the early stages, and atypical presentations are not common. Therefore, carefully integrating clinical courses, treatment history, laboratory data, and, when appropriate, bronchoalveolar lavage or histopathology, is essential. Only through a comprehensive evaluation can clinicians appropriately establish differential diagnoses and reliably exclude alternative etiologies before attributing findings to T-DXd-related ILD.

Corticosteroid responsiveness was generally favorable, consistent with the notion that inflammatory changes predominated at the onset of T-DXd-related ILD, whereas fibrotic changes were relatively uncommon. CT findings at presentation supported this interpretation, showing GGO and consolidation as the main features, with reticulation or traction bronchiectasis observed as minor changes. Preclinical data further reinforce this interpretation: an experimental study in cynomolgus monkeys demonstrated interstitial inflammatory changes in the lungs following exposure to T-DXd.^34^ Taken together, these observations suggest that inflammation is the principal pathology in the early phase of T-DXd-related ILD.

According to the T-DXd pulmonary toxicity management guidelines,^19^ for patients with Grade 3-4 ILD showing no clinical improvement within 3-5 days of corticosteroid initiation, additional evaluation for alternative etiologies and the use of immunosuppressants such as infliximab or mycophenolate mofetil should be considered, in accordance with local practice. In this report, immunosuppressive agents were administered to eight patients with T-DXd-related ILD; however, given the small number of cases and heterogeneity in treatment timing and regimens, a clear clinical benefit could not be determined. The agents used included cyclophosphamide, cyclosporine, azathioprine, tocilizumab, and tacrolimus, whereas infliximab and mycophenolate mofetil, explicitly recommended in the guideline,^19^ were not used.

This divergence likely reflects established practice patterns in Japan, where such agents have historically been used in the treatment of corticosteroid-resistant or corticosteroid-refractory idiopathic interstitial pneumonias.^35^ Furthermore, in these eight cases, the initiation of immunosuppressive therapy was not always performed strictly according to the recommendations of the T-DXd pulmonary toxicity management guidelines.^19^ Notably, the immunosuppressive agents introduced in the T-DXd pulmonary toxicity management guidelines were recommended for treating steroid-refractory or steroid-resistant pneumonitis induced by immune checkpoint inhibitors in the immune-related adverse event management guidelines.^36^ Although the efficacy of immunosuppressants was limited in our eight cases, recent literature has reported their potential clinical benefit in steroid-refractory T-DXd-related ILD. Emerging case reports and retrospective analyses have demonstrated successful management with various agents, including intravenous immunoglobulin,^37^ the antifibrotic agent nintedanib,^38^ cyclophosphamide,^39^^,^^40^ and combination therapy with infliximab and tacrolimus.^41^ As real-world experiences accumulate, it will be important to further evaluate the optimal timing and selection of these agents, as well as to consider revisions to current recommendations so that they better reflect actual clinical practice and optimize patient care.

The present report findings, together with current multidisciplinary guidance, highlight the importance of close monitoring, early detection, and prompt therapeutic intervention for patients treated with T-DXd, in accordance with the “Five S Rules” (Screen, Scan, Synergy, Suspend, Steroids) that have been proposed for the detection and management of T-DXd-related ILD.^42^ Although responsiveness to corticosteroids was observed in most cases, the higher mortality rate among patients with the DAD pattern and the limited data on off-label immunosuppressant use preclude any definitive conclusions regarding their clinical benefit in these patients. Our findings support a clinical rationale for prompt recognition of suspected T-DXd-related ILD and early consideration of corticosteroid therapy before progression to severe ILD such as DAD pattern, along with strict adherence to ILD management guidelines^19^^,^^43^; however, this should be interpreted as hypothesis-generating, as the present analysis was not designed to determine the optimal timing or regimen of corticosteroid treatment.

This report has several limitations. The all-patient PMS studies were conducted exclusively in Japan, and given that the Japanese population has a higher incidence of T-DXd-related ILD, caution should be exercised when generalizing these results to non-Japanese populations. As this analysis relied on post-marketing real-world data, the timing of imaging assessments was not standardized but left to the discretion of the treating physicians, which may have influenced the determination of ILD onset and outcome dates; this is an inherent limitation of PMS studies. Additionally, the sample size for certain subgroups, such as patients with Grade 4 ILD (*n* = 2), was too small to draw definitive clinical conclusions regarding corticosteroid responsiveness. Experience with the use of immunosuppressants in severe ILD cases remains limited. Ongoing accumulation of real-world cases, along with periodic reassessment of current management guidelines and exploration of new treatment strategies, is essential to further optimize the care of patients with T-DXd-related ILD.

## Conclusion

This report is significant given that it is the first to evaluate a large adjudicated real-world dataset of T-DXd-related ILD in a post-marketing setting in Japan. These findings support the theory/notion that the severity of T-DXd-related ILD is mild or moderate in most cases, and that appropriate interventions (such as corticosteroid administration) may lead to improvement of ILD in the majority of these non-fatal cases. However, they also reaffirm that cases exhibiting a DAD imaging pattern may be associated with poor prognosis, as the report identified 30 (representing 7.9% of all ILD cases) patients who died from the ILD despite receiving corticosteroid treatment. Although corticosteroid administration was generally effective, its use in these cases was heterogeneous and not always fully aligned with guideline-recommended regimens, highlighting the need for further accumulation of clinical data to establish optimal, standardized treatment strategies for T-DXd-related ILD. Nevertheless, despite the inherent variables in this real-world setting, our overall observations support the importance of vigilant monitoring, early recognition, and prompt corticosteroid treatment in accordance with current established guidelines to help mitigate fatal T-DXd-related ILD.^19^^,^^42^^,^^43^

## Supplementary Material

oyag340_Supplementary_Data

## Data Availability

The data included in this manuscript are used under a contract with the participating site, but the provision of data is not included in the contract, and they cannot be freely distributed by the authors.
