# Supplementary material for: Real-world characteristics of trastuzumab deruxtecan-related interstitial lung disease in Japanese patients with breast cancer and gastric cancer
Source: Oncologist. 2026 Sep 7;31(10):oyag340. doi: 10.1093/oncolo/oyag340 (PMC13653604; doi:10.1093/oncolo/oyag340)
Supplement: Supplementary file 1 [file oyag340_supplementary_data.zip › T-DXd-ILD-PMS_RQ1-MD_Supplementary_260817.docx]

**Supplementary Materials**

**Supplementary Figure S1.** Flow diagram of ILD cases

^a^Some cases were counted in more than one category.

^b^Unilateral pleuroparenchymal fibroelastosis following pleurodesis and passive atelectasis due to massive ascites.

^c^Three cases were not evaluable due to baseline, onset, or post-onset imaging data not being provided.

Abbreviations: AC, Adjudication Committee; BC, breast cancer; GC, gastric cancer; ILA, interstitial lung abnormalities; ILD, interstitial lung disease; PMS, post-marketing surveillance; T-DXd, trastuzumab deruxtecan.

**Supplementary Figure S2.** Imaging findings at ILD onset by lung lobe in T-DXd-related ILD cases (*N* = 377^a^) (**A**), by imaging pattern at the latest evaluation (**B**), and distribution of imaging findings at onset in T-DXd-related ILD by laterality and number of involved lobes (**C**)

^a^Of the 381 adjudicated T-DXd-related ILD cases, four cases in which chest CT images at or around ILD onset were not provided were excluded from the denominator.

^b^Some cases were counted in more than one category.

Abbreviations: CT, computed tomography; DAD, diffuse alveolar damage; GGO, ground glass opacities; HP, hypersensitivity pneumonitis; ILD, interstitial lung disease; OP, organizing pneumonia; T-DXd, trastuzumab deruxtecan.

**Supplementary Figure S3.** Use of immunosuppressants in T-DXd-related ILD

The figure is structured in four rows from top to bottom. The first row presents information related to ILD events. Black triangles (▲) indicate the adjudicated onset date of ILD, and the corresponding CTCAE grade at onset is displayed next to each ▲. If the adjudicated onset date differed from the onset date reported by the physician, the physician-reported onset date is indicated by white triangles (△) with CTCAE grade at onset determined by reporting physicians. Crosses (♰) indicate date of death due to ILD. The second row presents information on T-DXd administration. White bars represent the period of T-DXd treatment (from initial to the last administration), and the total number of T-DXd cycles administered is shown inside each bar. The third row presents corticosteroid treatment. High-dose corticosteroid therapy, defined as methylprednisolone ≥250 mg/day, is shown by black bars. Low-dose corticosteroid therapy, defined as methylprednisolone or prednisolone <250 mg/day, is shown by gray bars. The fourth row presents immunosuppressant use. Red bars represent periods of immunosuppressant therapy.

^a^The cases in which the imaging pattern changed during the course after the onset of ILD are indicated by arrows (→).

Each clinical course of ILD cases until outcome was summarized as below.

^b^Following the last dose (Day 295), Grade 1 ILD was diagnosed on Day 316. Despite PSL 35 mg/day from Day 323, patient deteriorated with rising KL-6. High-dose mPSL (1000 mg/day, 3 days) and azathioprine (4 days) started on Days 352–354 for worsening bilateral GGOs and respiratory distress requiring oxygen, but patient died on Day 355. ILD AC retrospectively adjudicated Grade 1 ILD onset on Day 182.

^c^The reporting physician diagnosed Grade 4 ILD on Day 323, the COVID-19 PCR test was positive, and tocilizumab was initiated. The ILD AC adjudicated to Grade 1 ILD on Day 259 (36 days before the last dose); the ILD AC concluded that, although the contribution of COVID-19 to the patient’s worsening condition and death could not be ruled out, the causality of T-DXd was strongly suspected.

^d^After the last dose (Day 295), the patient developed cough progressing to dyspnea. On Day 323, the patient was admitted with Grade 4 ILD and COVID-19 pneumonia (SpO_2_ 80% on oxygen 10 L). High-dose mPSL (1000 mg/day), DEX (6.6 mg/day), and tocilizumab (Day 325–326) were initiated. Despite oxygen support escalating to NHF and PSL taper, the patient died on Day 348 from acute respiratory deterioration. ILD AC retrospectively adjudicated Grade 1 ILD onset on Day 259.

^e^After the last dose (Day 190), the patient developed fever on Day 203, progressing to Grade 2 ILD on Day 206 with bilateral GGOs/dyspnea. PSL 50 mg/day was initiated; escalated to mPSL pulse on Day 208 for worsening oxygenation requiring NHF/NPPV. Despite intubation on Day 220 for severe hypoxemia and CY (500 mg) addition for poor steroid response, plus repeated pulses, the patient died on Day 251 from refractory respiratory failure.

^f^Following the last dose (Day 190), fever and cough developed on Day 203. Despite initial antibiotic treatment, Grade 3 ILD with typical DAD pattern was diagnosed on Day 210. Two courses of mPSL pulse therapy (days 210–212, 218–220) and CY (Day 221) were administered, but the patient's respiratory status progressively deteriorated despite oxygen therapy, resulting in death from Grade 5 ILD on Day 223.

^g^Following the last dose (Day 176), mild cough developed. Grade 2 ILD was diagnosed on Day 197 with worsening cough and mild dyspnea. mPSL (500 mg/day, Days 197–199; 1000 mg/day, Days 201–203) and CY (500 mg/day, Days 201–202) were administered. Despite NHF oxygen initiated on Day 202, respiratory status deteriorated, progressing to Grade 5 ILD and death on Day 207. ILD AC retrospectively adjudicated Grade 1 ILD onset on Day 155.

^h^Following the last dose (Day 106), dry cough developed. Grade 3 ILD with typical DAD pattern was diagnosed on Day 122 (SpO_2_ 84%). First mPSL pulse (1000 mg/day, Days 124–126) showed poor response. After transfer on Day 138, intubation was required (SpO_2_ 69%). Second mPSL pulse (1000>500>250 mg/day, Days 138–146), CY (500 mg, Day 140), cyclosporine (150 mg/day, Days 141–173), antibiotics, and escalating respiratory support were provided. Despite maximal treatment, the patient’s condition deteriorated with persistent bilateral GGO, progressing to Grade 5 ILD and death on Day 173.

^i^Imaging pattern could not be evaluated due to the absence of clinical or imaging data.

^j^Following the last dose (Day 43), fever and tachycardia developed on Day 64. Grade 2 ILD diagnosed on Day 71, progressing to Grade 3 on Day 74. First mPSL pulse (500 mg/day, Days 77–79) with antibiotics showed slow progression. Second mPSL pulse (500 mg/day, Days 89–91) was given. Due to persistent deterioration, tacrolimus (Days 103–118) was added to PSL. Oxygen therapy was required. Gradually improved with sequelae, discharged on Day 129 on HOT and PSL 20 mg/day.

^k^Following the last dose (Day 22), Grade 3 ILD with DAD pattern developed on Day 43 (fever 39℃, bilateral infiltrates, right-dominant GGO, pleural effusion). Two mPSL pulses (1000 mg/day, Days 44–46, 50–52) with PSL and antibiotics were administered. Due to insufficient response after the second pulse, CY was added (500 mg/day, administration day not provided). Despite BiPAP support and treatments, the patient’s condition deteriorated, progressing to Grade 5 ILD and death on Day 67.

Abbreviations: AC, Adjudication Committee; BiPAP, Biphasic Positive Airway Pressure; CTCAE, Common Terminology Criteria for Adverse Events; CY, cyclophosphamide; DAD, diffuse alveolar damage; DEX, dexamethasone; F, female; G, Grade; GGO, ground glass opacities; HOT, home oxygen therapy; HP, hypersensitivity pneumonitis; ILD, interstitial lung disease; KL-6, Krebs von den Lungen-6; M, male; mPSL, methylprednisolone; NHF, nasal high flow; NPPV, non-invasive positive pressure ventilation; NSIP, non-specific interstitial pneumonia; OP, organizing pneumonia; PCR, polymerase chain reaction; PSL, prednisolone; T-DXd, trastuzumab deruxtecan.
